# Role of Anti-coagulation in Lemierre's Syndrome With Empyema

**DOI:** 10.7759/cureus.92256

**Published:** 2025-09-14

**Authors:** Lwin Paing, Khalid Shahzad, Thin Hlaing, Thi Htwe, Saquib Siddiqui

**Affiliations:** 1 Respiratory Medicine, James Cook University Hospital, Middlesbrough, GBR; 2 Respiratory Medicine, Queen Elizabeth Gateshead Hospital, Newcastle Upon Tyne, GBR; 3 Acute Medicine, York and Scarborough Teaching Hospital, York, GBR; 4 Respiratory Medicine, James Cook University Hospital, Newcastle Upon Tyne, GBR

**Keywords:** anti-coagulation, dental abscess, empyema, lemierre's syn, supra-clavicular abscess, thrombosis of the internal jugular vein

## Abstract

Lemierre's syndrome (LS) is a rare and potentially life-threatening condition that arises as a complication of oropharyngeal infections, characterized by internal jugular vein thrombosis and septicemia. This report describes a rare case of a 26-year-old previously healthy man with typical manifestations mentioned above and additionally with empyema. Management approach was multidisciplinary inputs comprised of antibiotics and surgical measures including anti-coagulation under a specialist’s opinion; nonetheless, it has been a debated issue. The patient improved clinically with normalization of inflammatory markers and radiological resolution of the effusion. He was discharged with a six-week antibiotic course and transitioned to rivaroxaban. This case highlights the importance of early recognition and multidisciplinary management of LS with thoracic complications.

## Introduction

Lemierre's syndrome (LS) is a rare and potentially life-threatening septic thrombophlebitis of the internal jugular vein, leading to thromboembolism and septicemia as a consequence of oropharyngeal infection, commonly caused by Fusobacterial infection. Diagnosis is often elusive on initial presentation. It should be suspected in a young, healthy patients exhibiting prolonged pharyngitis symptoms, followed by signs of septicemia, pneumonia, and unusual neck pain or swelling. Diagnosis is typically confirmed by identifying internal jugular vein thrombophlebitis and anaerobic growth in blood cultures. Commonly identified responsible organisms are *Fusobacterium necrophorum*, an obligate anaerobic, gram-negative bacilli, and *Fusobacterium nucleatum*. Other less common ones are Streptococcus species, Bacteroides species, *Staphylococcus aureus*, and *Klebsiella pneumonia* [1]. It is important to emphasize that a negative blood culture result does not entirely exclude LS [2].

Generally, antibiotic is the mainstay of treatment alongside source control. Anti-coagulation therapy in LS is controversial. Thrombus typically resolves independently. There are no controlled trials to guide us probably due to the rarity of the disease spectrum. On review of retrospective studies, contradicting statements were observed regarding the outcomes of anticoagulation which questioned the role of anticoagulation. This created a challenge for clinicians whether to anti-coagulate or not in LS. This report discusses a rare presentation of LS complicated by bilateral empyema and explores the therapeutic dilemmas faced in management.

## Case presentation

A 26-year-old man presented to the emergency department with a five-day history of feeling unwell with vomiting, followed by the development of a neck lump and difficulty breathing. He was generally fit and well with no comorbidities. He gave a history of recent dental abscess a week prior to developing a neck lump. According to the patient, he had three cracked teeth and abscess. There was no history of recreational drug misuse.

On examination, he had a left supraclavicular lump with tenderness. Overlying skin was hot and erythematous. On chest auscultation, he had reduced air entry more on the right side. Initial laboratory results revealed raised inflammatory markers (Table 1). Blood culture was sent, which showed positivity for *Fusobacterium necrophorum*.

**Table 1:** Laboratory findings

Lab Test	Value	Reference Range
White blood cell count	22.5	4.0-11.0 x 10^9^/L
Neutrophil count	18.5	1.5 to 8.0 x 10^9^/L
C-reactive protein	284	0-5 mg/l

**Figure 1:**
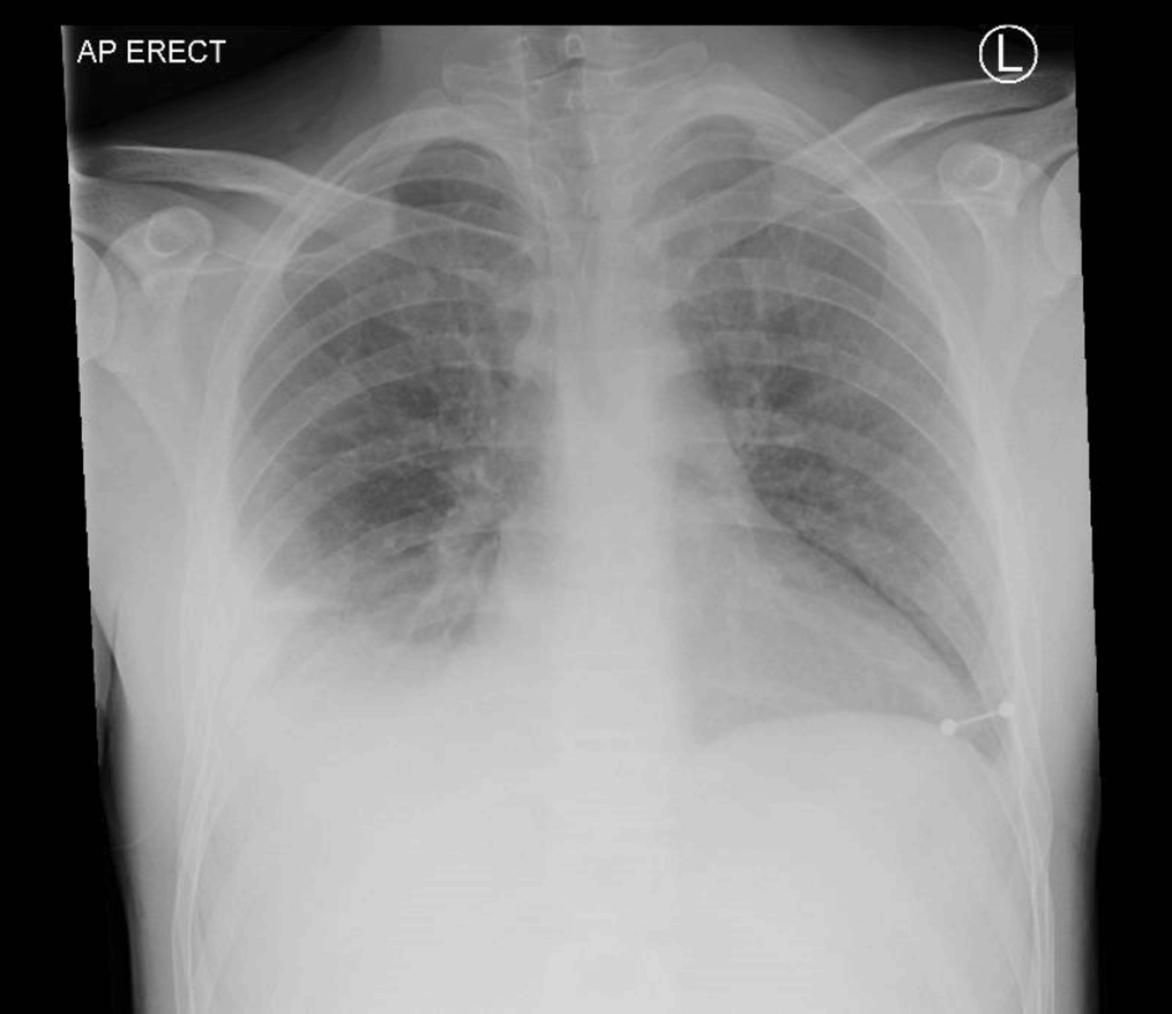
Chest X-ray showing significant costophrenic angle obliteration and opacification in the right lower zone and minimal on the left lower zone.

**Figure 2:**
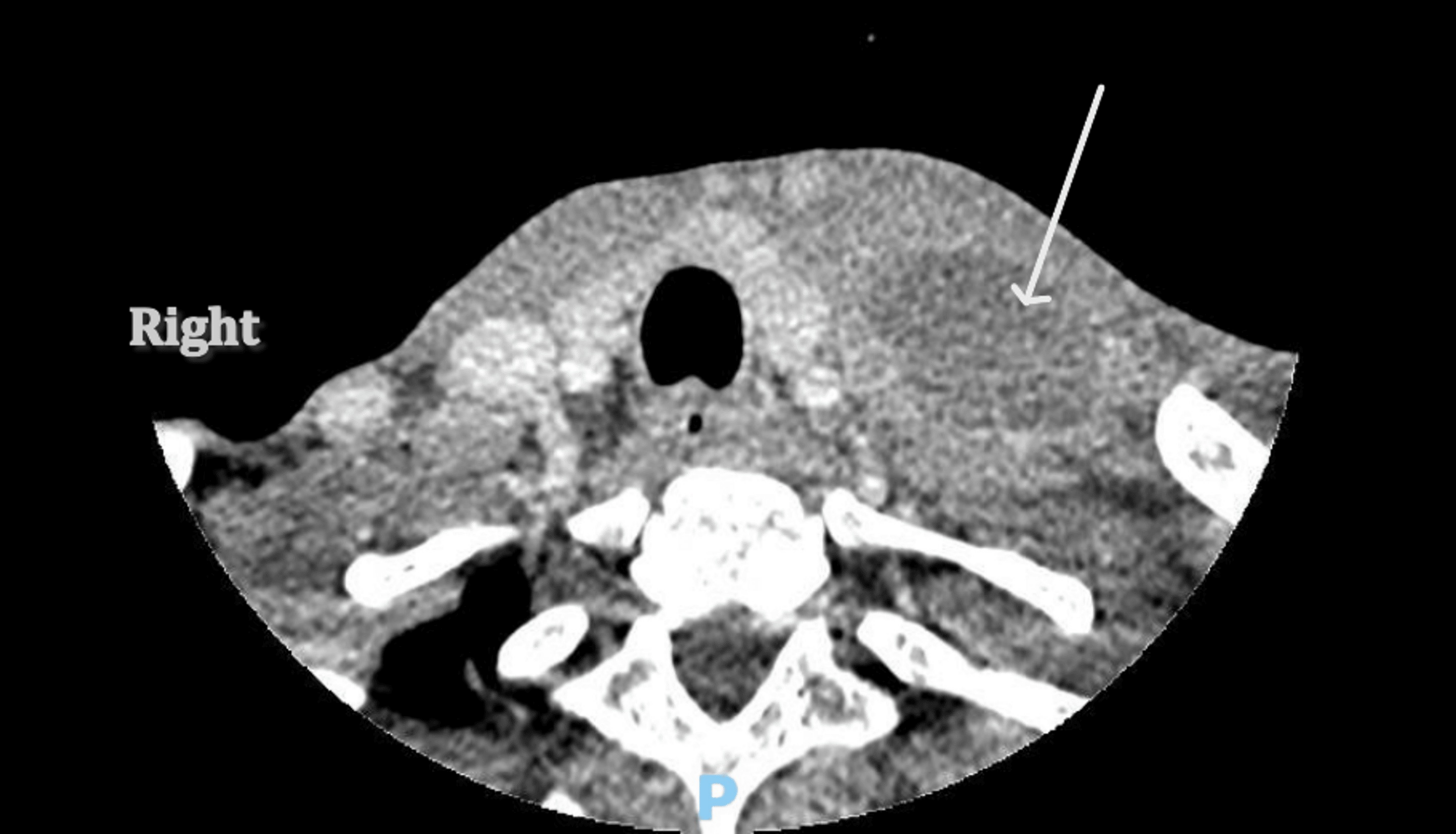
CT of the neck showing left supraclavicular abscess The arrow points to a well-defined, low-attenuation (hypodense) fluid collection as neck abscess on the left side.

**Figure 3:**
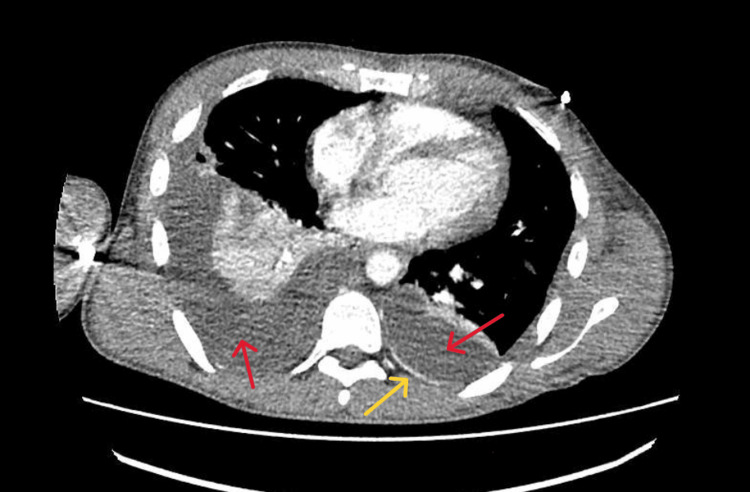
CT of the chest showing bilateral pleural effusion as low attenuation on both sides of the lung (red arrows), along with rim hyperattenuation as pleural splitting sign (pointed by yellow arrow)

An X-ray chest performed in the emergency department revealed bilateral pleural effusion, with the larger one on the right side (Figure 1). CT of the neck (Figure 2) and CT of the chest (Figure 3) revealed a left supraclavicular abscess extending into the thoracic inlet, with left internal jugular vein thrombosis. Bilateral effusion was also noted.

Bedside thoracic ultrasound revealed complex, large right-sided effusion. Diagnostic pleural tap revealed pus with pH 6.9.

He was started on broad-spectrum antibiotics (ceftriaxone and metronidazole). Therapeutic conundrum centered around anti-coagulation, as considering internal jugular vein thrombus, he required therapeutic low-molecular-weight heparin, but drainage of neck abscess and thoracostomy were also essential. Therefore, the case was discussed with the hematology team, which recommended to only give prophylactic low-molecular-weight heparin till the patient undergoes drainage of the neck abscess and insertion of an intercostal drain.

Ultrasound-guided drainage of the neck abscess was performed. Sample showed acute-on-chronic inflammation with granulation tissue. An 18-French intercostal chest drain was inserted on the right side.

Since dental abscess with three cracked teeth was imperative to manage, the dental team reviewed him and later performed extraction of the affected teeth. Till then, he was given a prophylactic dose of anti-coagulation. After teeth extraction, he was switched to therapeutic anti-coagulation. The neck lump resolved following drainage but had incomplete drainage of the right-sided empyema despite the 18-French intercostal drain.

As he was being treated with therapeutic dose low-molecular-weight heparin, intrapleural fibrinolysis was considered to be unsafe with high bleeding risk. Therefore, he was referred to the cardiothoracic team for video-assisted thoracoscopy (VATS) washout. As the current chest drain was unable to drain the effusion fully, there was no reason to continue with it, and only antibiotics was unlikely to resolve the empyema completely without further drainage of the remaining fluid. Consideration was made for a surgical drain insertion, but VATS washout seemed to be the more efficient choice and more likely for a rapid and safe discharge from the hospital. His inflammatory markers returned to normal levels after a week of intravenous antibiotics. The dental and ENT teams were satisfied with the progress. His imaging showed resolution of the effusion, and bedside thoracic ultrasound only showed consolidated lungs with minimal fluid.

Outcome and follow-up

He was discharged with a total six-week course of antibiotics after discussion with the microbiologist and the infectious disease team. Low-molecular-weight heparin was switched to rivaroxaban for 12 weeks on discharge.

## Discussion

LS is infrequently seen by general physicians. It was initially described by the French bacteriologist Andre Lemierre nearly a century ago [1]. He documented 20 cases of anaerobic septicemia originating from infections in the oropharynx [2]. The infection spreads into the lateral pharyngeal spaces of the neck, leading to a neck abscess and septic thrombophlebitis of the internal jugular vein(s) [3,4]. Although rare, it is typically considered a life-threatening condition. Patients with LS are at a significant risk of thromboembolic complications and death. A study by Valerio et al. involving 712 patients with LS found that 84% developed head and neck thrombosis and 82% had septic emboli, with 80% experiencing both. Despite hospital treatment with antibiotics and anti-coagulation, 7.2% of patients developed new venous thromboembolism, and 17.3% of patients had a new peripheral septic embolus [5].

The most commonly identified organisms responsible for the condition are *Fusobacterium necrophorum*, an obligate anaerobic, gram-negative bacillus, and *Fusobacterium nucleatum*. Less common organisms include Streptococcus species, Bacteroides species, *Staphylococcus aureus*, and *Klebsiella pneumoniae *[6-8].

Our patient initially had a dental infection that spread to the neck, resulting in a neck abscess and left internal jugular vein thrombophlebitis with thrombosis. The infection further spread to the pleural cavity, leading to empyema. Interestingly, the patient had bilateral empyema, which has not been previously reported in cases of LS. To the best of our knowledge, this is the first report of bilateral empyema in LS.

A bacterial pathogen may be identified from blood cultures, but a negative result does not rule out the condition, as there have been cases of LS without positive blood cultures [9]. Notably, *Fusobacterium necrophorum*, the most frequently identified organism, can take up to seven days to grow in blood culture and may be mistaken for Bacteroides species [10,11]. Antibiotics are the mainstay of treatment along with source control. Based on the culture results, we treated our patient with ceftriaxone and metronidazole. Microbes are generally resistant to macrolides and even more so to gentamicin and quinolones. Therefore, most experts recommend antibiotics targeting anaerobes, such as a combination of metronidazole and penicillin or cephalosporin [10,12]. We referred the patient to the dental team, where all affected teeth were extracted.

The use of anti-coagulation therapy in LS is controversial. Thrombi typically resolve on their own, and no controlled trials have been conducted to guide treatment, likely due to the rarity of the condition. Literature reviews revealed that uncomplicated cases without extensive clot burden generally do not require anti-coagulation and can be effectively treated with antibiotics. In 2015, a retrospective study by Cupit et al. found no difference in outcomes between patients treated with or without anti-coagulation [13]. However, the study only included patients with internal jugular vein thrombosis, not extensive clot burden. It is reasonable to assume that patients with limited clot burden can be treated without anti-coagulation. Similar findings were noted in a study by Nygren et al. [14].

Our patient did not have extensive clots outside the internal jugular vein, raising questions about the need for anti-coagulation. After consulting with our hematology team, we decided to proceed with anti-coagulation, referencing the study by Valerio et al., which found that anti-coagulation was associated with fewer new thromboembolic events or peripheral septic emboli [5]. The study showed that 34 out of 712 patients developed new VTE, with 65% not receiving anti-coagulation compared to 35% who did. Among 76 patients who developed new peripheral septic emboli, 61% were not treated with anti-coagulation versus 39% who were. Only 2.8% (19 out of 398 patients) of those treated with anti-coagulation experienced major bleeding, and 8 of those 19 had thrombocytopenia. Although the study did not specify platelet counts, the general guideline is that anti-coagulation is safe above 50,000. Considering the study, our team felt that initiating anti-coagulation would be in the patient's best interest to prevent further VTE. There is no standard recommendation for the duration of anti-coagulation. Adedeji et al. recommended that a duration of 6 to 12 weeks is safe and effective [15].

We chose to insert an 18-French intercostal chest drain on the right side, as it was larger, and the patient was not willing for simultaneous drain or aspiration. Despite five days of drainage, we were unable to achieve a complete resolution. A bedside thoracic ultrasound and chest CT scan confirmed the presence of a complex pleural effusion. Intrapleural thrombolysis was considered given its established role in pleural infection and empyema. However, concomitant use of therapeutic anticoagulation with MIST-2 protocol or intrapleural thrombolysis is associated with an increased risk of bleeding (9.6%). In this case, there was concern that interruption of therapeutic anticoagulation might exacerbate the existing internal jugular vein thrombosis or lead to the formation of new thrombi [16]. Therefore, we consulted our cardiothoracic team for surgical intervention. The patient subsequently underwent a thoracotomy with pleural decortication. We eventually discharged the patient with a six-week course of antibiotics and a 12-week course of anti-coagulation, achieving a satisfactory clinical outcome during outpatient follow-up.

Patients with LS can present with complex therapeutic challenges beyond antibiotic and anti-coagulation treatment. These patients are generally young adults, and clinicians should maintain a high index of suspicion. The best diagnostic method for confirming internal jugular vein thrombosis is a contrast-enhanced CT scan showing luminal filling defects or thrombus formation. Once septic thrombophlebitis occurs, potential complications such as pneumonia, empyema, intracranial abscess, or venous thromboembolic disorders should be evaluated. Successful management should involve a multidisciplinary approach and appropriate antibiotic therapy alongside anti-coagulation, although further clarification is needed regarding the role and duration of anti-coagulation.

## Conclusions

LS is a rare but potentially fatal condition requiring early recognition and prompt treatment. Our case highlights the classical features with the unusual occurrence of bilateral empyema. Management involves prolonged antibiotic therapy and timely source control. The role of anti-coagulation remains controversial. However, in this case, it was initiated following multidisciplinary input due to the risk of thromboembolic complications. Individualized assessment remains key, as further evidence is needed to guide anti-coagulation use and duration in LS.
